# Addressing Parental Vaccine Hesitancy and Other Barriers to Childhood/Adolescent Vaccination Uptake During the Coronavirus (COVID-19) Pandemic

**DOI:** 10.3389/fimmu.2021.663074

**Published:** 2021-03-18

**Authors:** Olufunto A. Olusanya, Robert A. Bednarczyk, Robert L. Davis, Arash Shaban-Nejad

**Affiliations:** ^1^ Oak Ridge National Laboratory, Center for Biomedical Informatics, Department of Pediatrics, College of Medicine, University of Tennessee Health Science Center, Memphis, TN, United States; ^2^ Hubert Department of Global Health, Rollins School of Public Health, Emory University, Atlanta, GA, United States

**Keywords:** vaccine hesitancy, vaccine confidence, vaccine compliance, vaccine disparity, COVID-19, SARS-COV-2, Social Determinants of Health (SDoH)

## Abstract

Routine childhood immunizations are proven to be one of the most effective public health interventions at controlling numerous deadly diseases. Therefore, the CDC recommends routine immunizations for children and adolescent populations against vaccine-preventable diseases e.g., tetanus, pertussis, diphtheria, etc. This current review sought to examine barriers to pediatric vaccine uptake behaviors during the COVID-19 pandemic. We also explored the implications for parental vaccine hesitancy/delay during an ongoing health crisis and proposed recommendations for increasing vaccine confidence and compliance. Our review determined that the receipt for vaccinations steadily improved in the last decade for both the United States and Tennessee. However, this incremental progress has been forestalled by the COVID-19 pandemic and other barriers i.e. parental vaccine hesitancy, social determinants of health (SDoH) inequalities, etc. which further exacerbate vaccination disparities. Moreover, non-compliance to routine vaccinations could cause an outbreak of diseases, thereby, worsening the ongoing health crisis and already strained health care system. Healthcare providers are uniquely positioned to offer effective recommendations with presumptive languaging to increase vaccination rates, as well as, address parental vaccine hesitancy. Best practices that incorporate healthcare providers’ quality improvement coaching, vaccination reminder recall systems, adherence to standardized safety protocols (physical distancing, hand hygiene practices, etc.), as well as, offer telehealth and outdoor/drive-through/curbside vaccination services, etc. are warranted. Additionally, a concerted effort should be made to utilize public health surveillance systems to collect, analyze, and interpret data, thereby, ensuring the dissemination of timely, accurate health information for effective health policy decision-making e.g., vaccine distribution, etc.

## Introduction

In the United States (U.S.) and globally, routine prophylactic childhood immunizations are established as public health interventions that are most effective and cost-beneficial at significantly preventing numerous infectious diseases and premature mortalities (1). In the pre-vaccine era and before the 1963 measles vaccination programs, there were roughly 6,000 deaths attributed to the measles virus each year (2). Moreover, between 1964 and 1965, an epidemic of the rubella virus resulted in an estimated 2,000 neonatal deaths and 11,000 fetal miscarriages (3). Accordingly, it has been projected that over 100 million cases of vaccine-preventable illnesses i.e. measles, mumps, rubella, pertussis, etc., have been prevented in the U.S. (4). Between 1994 and 2013, an enormous financial burden to the tune of approximately $402 billion and $1.5 trillion was prevented in direct and societal costs (5). Concurrently, within Tennessee, a racially and economically diverse state ranking 16^th^ most populous in the U.S, vaccine-preventable diseases have significantly decreased. This is due to widespread institutional policies to increase vaccine uptake (to meet Tennessee Immunization Program (TIP)’s 90% goal), as well as, wider acceptance of healthcare providers’ recommendations (6). Overall, vaccinations continue to serve an essential role in protecting vulnerable individuals from potentially deadly vaccine-preventable illnesses.

Moreover, the scientific community supports the consensus that the highly contagious coronavirus disease 2019 (COVID-19), which was declared a pandemic by the World Health Organization (WHO) in March 2020, can be controlled with an effective COVID-19 vaccine (7). The COVID-19 presents with a continuum of respiratory tract symptoms such as fever, shortness of breath, pneumonia, influenza-like illness, etc., and is caused by the Severe Acute Respiratory Syndrome Coronavirus 2 (SARS-COV-2) (8, 9). In the U.S., the COVID-19 has resulted in 28,405,925 incident cases and 511,839 mortalities. Whereas, in Tennessee, 2.7% of total U.S. cases (775,693) and 2.2% of total U.S. deaths (11,421) have been recorded (10). Following mutations in their virus genome, new variants of the SARS-COV-2 have begun to emerge with alterations to their features. Concerningly, these genetic variants may increase disease severity and infectivity as well as change treatment and vaccine efficacy (11). Consequently, this ongoing public health crisis from COVID-19 has had devastating impacts on *every* aspect of human life causing significant morbidity and mortality, adverse psychological outcomes, and growing socioeconomic losses. Additionally, the pandemic has disrupted the hard-earned progress made in the last decade to improve vaccination rates. This current review sought to explore the barriers to pediatric vaccine uptake behaviors (e.g., vaccine hesitancy), as well as, propose recommendations for increasing vaccine confidence and compliance to immunization schedules within the context of the COVID-19 pandemic.

## Vaccination Coverage Among Children (19–35 Months) and Adolescents (13–17 Years) in Tennessee and the U.S.

**Figure 1:**
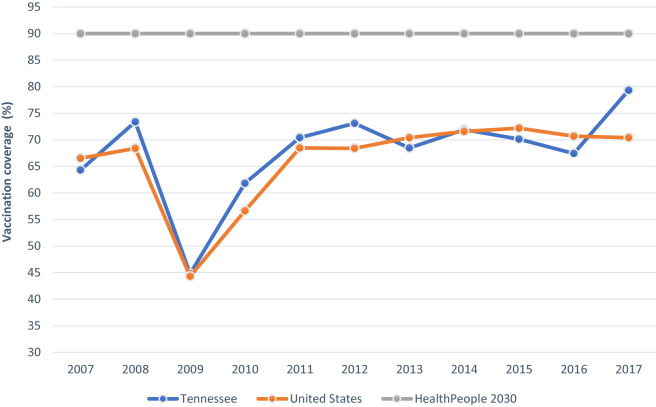
Combined 7-Vaccine Series Coverage (%) by Year among Children ages 19-35 months in Tennessee and the United States in Relation to the HealthyPeople2030 Goal.Combined 7-vaccine series: ≥4 doses of diphtheria, tetanus toxoid, and pertussis, ≥3 doses of polio, ≥1 measles-containing vaccine, influenza 1b full series, ≥3 hepatitis b, ≥1 varicella and ≥4 pneumococcal vaccine. Data source: National Center for Immunization and Respiratory Diseases. Retrieved October 13, 2020

The CDC’s Advisory Committee on Immunization Practice (ACIP) recommends routine immunizations against diseases e.g., measles, whooping cough for children ages through 2 years. For the 78.6 million children born between 1994 and 2013 in the U.S., routine childhood vaccinations have prevented an estimated 322 million illnesses, 21 million hospitalizations and 732,000 untimely deaths from measles (70,748), varicella (68,445), pertussis (54,406), mumps (42,704), and rubella (36,540) (10). Consequently, within the last decade i.e. 2009-2017, coverage for the combined 7-vaccine series^1^ among children ages 19-35 months has risen comparably in the U.S. and Tennessee from 44.3% to 72.2% and 44.8% to 79.3%, respectively (12) (**Figure 1**).

**Figure 2:**
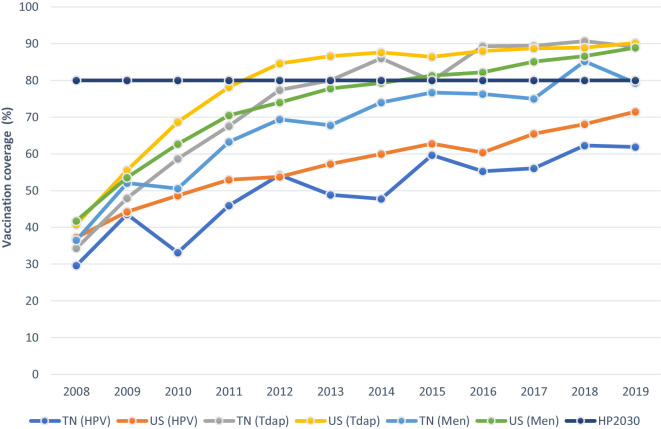
Estimated Vaccine Coverage (%) by Year among Adolescents ages 13-17 years in Tennessee and the United States in Relation to the HealthyPeople2030 Goal.HPV= human papillomavirus vaccine; Tdap= tetanus toxoid, reduced diphtheria toxoid, and acellular pertussis vaccine; Men= meningococcal conjugate vaccine, TN=Tennessee, US=United States. HPV, Tdap, and meningococcal conjugate vaccinations are depicted as the receipt of ≥1 dose of vaccine Data source: National Center for Immunization and Respiratory Diseases. Retrieved October 13, 2020

Concomitantly, among adolescents, vaccinations are recommended to prevent illnesses such as human papillomavirus (HPV), whooping cough, and meningococcal disease. As portrayed in **Figure 2**, HPV coverage; diphtheria, tetanus, and acellular pertussis (Tdap); and meningococcal conjugate vaccines have steadily increased in the last decade within the U.S. and Tennessee (12). Although, while the prevalence for all specified vaccines varied slightly within a 10%-point range in 2008, HPV vaccination rates have continued to significantly lag behind that of Tdap and meningococcal conjugate vaccines in recent years. While national vaccination rates for Tdap and meningococcal conjugate vaccines have reached or exceeded the HealthyPeople 2030 set-goal of 80% for vaccine coverage among adolescents (13 to 17 years), immunization with the HPV vaccine remains considerably low. See **Figure 2**. Despite current ACIP protocols and improving trends for other vaccines, the 2019 coverage for ≥1 HPV vaccine among male and female adolescents was estimated at 71.5% in the US and 9.6 percentage points lower (61.9%) in Tennessee (13).

## Barriers to Childhood/Adolescent Vaccination Uptake During the COVID-19 Pandemic

### Impacts of the COVID-19 Pandemic on Vaccination Rates

As part of the efforts to “flatten the curve” and control the rapid spread of SARS-COV-2 during the COVID-19 pandemic, numerous policies and preventive public health measures including shelter-in-place, stay-at-home orders, social distancing, lockdowns, and other quarantine measures, were imposed (13). These precautionary measures, which have disrupted healthcare systems and health personnel services, have ultimately led to sub-optimal vaccine delivery services and vaccination rates (14). In the U.S., after the national emergency declaration, the aggregate count for pediatric vaccine doses procured by the Vaccine-for-Children (VFC) providers substantially declined (15). Similarly, the WHO recorded a 28-year reduction in global coverage for the Tdap vaccine (16). Also, notable was the collective shift in focus on routine vaccinations to respond urgently to the ongoing health crisis (17). These events have been exacerbated by the adverse rippling effects of other COVID-19 pandemic sequelae which include overwhelmed healthcare systems; inequalities in healthcare delivery; financial recession and job losses; worries about vaccine costs; inadequate personal protective equipment for healthcare workers; severe shortages in testing modalities and treatment therapies; long-term school closures; contradictory messages from health agencies/authorities; as well as; disruptions to transportation and travel restrictions. Additionally, parental concerns regarding exposure to the COVID-19 have discouraged individuals who would otherwise have utilized vaccination services, thus, resulting in postponed/canceled medical appointment visits. Moreover, restrictions on routine in-person office visits due to physical distancing protocols have limited health care providers’ communications promoting vaccine uptake to patients (18).

Overall, the existing COVID-19 pandemic has forestalled the painstaking but incremental progress made in the last decade to improve uptake for HPV and other vaccines. Disruptions to vaccine delivery services have negatively impacted timely immunizations leaving children/adolescents susceptible to vaccine-preventable diseases. As the United Nations Children’s Fund (UNICEF) Executive Director Henrietta Fore aptly describes it, “COVID-19 has made previously routine vaccination a daunting challenge…” (16). An outbreak of vaccine-preventable diseases during the COVID-19 pandemic would only worsen the already strained health care system due to rising hospitalization and death rates.

### Impacts of Social Determinants of Health on Vaccination Rates

In research, individual and interpersonal level approaches have long been utilized to examine and facilitate behavior change. However, this approach is limited as it fails to integrate societal components that influence health outcomes. More recently, factors that facilitate and/or hinder the implementation of health behaviors are addressed using a novel approach - Social Determinants of Health (SDoH) (19). The SDoH encompasses elements in an individual’s neighborhood, community, and environment as determined by where that individual is born, resides, learns, works, worships, etc. Consequently, the HealthyPeople 2030 SDoH Framework classifies SDoH indicators into five categories: social and community context, education, economic stability, neighborhood, and built environment, and health and health care (19). SDoH include access to education, affordable housing and health services, public safety, food security, etc. (20).

SDoH are impacted by the distribution of resources that improve the quality of life and public health outcomes. For instance, in the U.S., individuals who reside in certain metropolitan statistical areas (MSA), non-MSA (mostly rural), and without health insurance are disproportionately less likely to be vaccinated (21). Additionally, parental education; household living conditions and income; healthcare access; philosophical and cultural beliefs; religious affiliations; and urban Vs. rural residence, are some of the SDoH that influence childhood vaccination rates (22). In recent times, the COVID-19 pandemic has underscored the importance of incorporating SDoH into health systems and health service delivery. While only a few studies have examined the impacts of SDoH on vaccinations, it is likely the COVID-19 pandemic has exacerbated the adverse effects of some SDoH on vaccination uptake behaviors e.g., employment, poverty, healthcare access, food insecurity, education, etc.

### Impacts of the Vaccine Hesitancy/Refusal on Vaccination Rates

The WHO describes vaccine hesitancy as the, “delay in acceptance or refusal of vaccines despite availability of vaccine services” and categorizes it within the top ten threats to global health (23). This phenomenon also incorporates the antivaccine movement as well as parents’ adoption of alternate, non-standardized vaccination schedules. Parent’s hesitancy, refusals, and delays in adhering to routine childhood immunizations are largely responsible for a significant number of unvaccinated/under-vaccinated children, disease outbreaks, co-morbidities (e.g., meningitis, pneumonia, HPV-related cancers), as well as, untimely deaths. Vaccine hesitancy and refusal have mostly occurred due to state/local policies that have allowed parents to decline routine childhood vaccinations based on non-medical exemptions (24). These non-medical exemptions occur in the form of religious exemptions e.g., due to an individual’s religious beliefs which oppose the use of fetal tissue for vaccines and personal belief exemptions e.g., due to an individual’s logical reasoning which disapproves the use of non-natural products for vaccines (24). Prevalence estimates for vaccination exemptions are currently 2.5% and 1.9% nationally and in Tennessee, respectively (25). Currently, in the U.S., 45 states and Washington D.C. permit religious exemptions while 15 states allow philosophical exemptions from childhood vaccinations.

Due to parental concerns on vaccine safety/side effects, some studies which implied a link between the measles-mumps-rubella (MMR) vaccine and autism, played a significant role in vaccine hesitancy and refusal (26, 27). However, Wakefield et al. was retracted due to methodological deficits and data misrepresentation (28). Following larger multiple studies and a wealth of scientific evidence, this hypothesized link was disproved and the safety of the MMR vaccine reinforced (29, 30). Likewise, fears regarding administering multiple vaccines concurrently in a child, unverified sources, and misinformation campaigns from the internet/media have served to dissuade parents from seeking child vaccination services (22). Nevertheless, evidence-focused literature has debunked numerous myths and misinformation citing that recommended vaccines are too many; contain unfavorable ingredients e.g., mercury, aluminum, DNA fragments; damage immune and neurologic systems; and display life-threatening side-effects, etc. (31).

## Implications of Non-Adherence to Vaccination Protocols

Overall, a major accomplishment of universal vaccine coverage has been to markedly reduce and/or eradicate transmittable diseases that would ultimately have led to premature mortalities in the pre-vaccine era. Despite these advances, however, sporadic outbreaks within communities have continued to occur and coincide with pockets of low community vaccination rates and limited ability for vaccines to elicit immune responses (32). The majority of recent outbreaks have occurred among unvaccinated individuals particularly those exposed to illnesses imported from other countries, as well as those who claimed religious or personal exemptions or had missed immunization opportunities (33, 34).

As a result, outbreaks, incidence, prevalence, and transmission of illnesses e.g., measles virus are seeing an increasing trend in the U.S. (35). Between January and December 2019, there were 1,282 confirmed cases of measles reported in 31 states. This is significantly higher than the 375 cases seen in 2018 and represents the highest prevalence reported since 1992 (36). Moreover, in 2019, almost half of the 14 counties that granted non-medical vaccination exemptions to parents of kindergarten school-aged children (37) experienced the measles outbreak (38). Accounts of other vaccine-preventable outbreaks have occurred for the Haemophilus influenza type B (39) and pneumococcal infections (40).

In addition, adolescents engaging in risky sexual behaviors e.g., multiple sexual partners, and unprotected sexual intercourse, are susceptible and considered high risk for acquiring HPV infections (41). Between 2013 and 2017, there were an estimated 45,300 HPV-associated cancers recorded consisting of cervical (12,143), oropharyngeal (19,775), and anal (7,083) cancers (42). More than 90% of all HPV-associated cancers (e.g., cervical, vulvar, vaginal, and anal cancers) are preventable through receipt of the HPV vaccine (43).

## Recommendations to Restore Parental Vaccine Confidence during the COVID-19 Pandemic

Despite the disruption to health amenities during the COVID-19 pandemic, the continuity of immunization services for children and adolescents is pertinent to enable progress in vaccination trends as well as deter vaccine-preventable diseases and outbreaks. Against the backdrop of the COVID-19 pandemic and vaccine non-compliance/refusal, the pediatrician and other healthcare providers are uniquely qualified to promote vaccinations achieved through the use of strong, presumptive languaging (44) to offer effective, consistent recommendations that emphasize disease/cancer prevention. The healthcare professionals’ reluctance to share recommendations that facilitate vaccine uptake could result in parental hesitancy, refusal, and delay. In 2019, national HPV coverage among adolescents with a provider’s recommendation (74.7% CI:73.3-76.0) was almost twice as those without one (46.7% CI:43.8-49.6) (21). Concurrently, in Tennessee, coverage was 72.4% (CI:64.3-79.2) for those who received advice from their providers as opposed to those without (28.2% CI:16.6-43.6) (21). This supports the notion that recommendations offered by health providers could significantly predict vaccine uptake, thereby reinforcing the need for personalized patient-provider interactions.

Consequently, interventions and training should empower healthcare providers to disseminate evidence-based advice on vaccines. Specifically, quality improvement coaching such as the CDC’s Assessment, Feedback, Incentives, and eXchange (AFIX) program which facilitates provider’s education and feedback through face-to-face coaching has been shown to improve immunization rates (45). Also, campaigns should aim to increase providers’ self-efficacy and confidence to address parental concerns on vaccine’s efficacy, side effect(s), lack of health insurance as well as adopt the use of electronic medical records (EMRs), immunization information systems, and medical practice alerts to remind parents about scheduled regular in-patient visits (46). Parents without health insurance should receive information on reduced out-of-pocket costs and publicly-funded vaccines available through the VFC program (13). For parents with religious or philosophical beliefs, healthcare provider’s information on the fewer components of proteins and polysaccharides in vaccines could serve to allay fears (38). For others, communication on societal norms that promote routine vaccination as a social responsibility could increase vaccine uptake (47). Moreover, addressing parental concerns for needle pain, skin reactions, and sensitivity as well as the adoption of motivational interview techniques (i.e. acceptance, compassion, collaboration, etc.) could be impactful (38).

Furthermore, best-practices that facilitate adherence to standardized safety protocols, beneficence, and non-transmission of the COVID-19 should be employed e.g., physical distancing, mask usage, hand hygiene practices, etc. Training and instructions on disease/infection prevention and control should be incorporated into the continuing medical education (CME) curriculum for health professionals (48). Wellness-child visits through telemedicine video conferencing; administering of vaccines through outdoor/curbside/drive-through services; vaccine delivery in alternative settings e.g., pharmacies, schools; minimizing on-site patient visit at any single point in time; delineating specific, well-ventilated rooms for wellness visits, vaccine-only visits, etc., should be implemented to tackle the current COVID-19 health crisis (14). Overall, clinicians should work in synergy with other healthcare team members to maximize scheduled wellness/immunization visits, and other routine medical checkups particularly in places with a low prevalence of health provider’s recommendations e.g., rural areas. While mandatory vaccination policies have been shown to be associated with higher vaccine acceptance rates (49), these should be reinforced with patient-provider interactions that address parental concerns. Additionally, nonmedical exemption laws should be reviewed to ensure that in places where they have not been prohibited, there should be in place effective administrative controls so that exemptions do not become easier defaults when compared to vaccinations (50, 51). Government health officials, as well as, the school districts should continue to enforce and maintain up-to-date immunization records. The catch-up vaccination protocols issued by the CDC to facilitate coverage for children with missed appointments during the pandemic should be implemented (52). Education campaigns should also be tailored to engage local and religious leaders, be culturally appropriate and address specific concerns from vaccine-hesitant populations.

Ultimately, a multifaceted, multidisciplinary approach involving science, engineering, and social sciences should be incorporated to explore facilitators and barriers to childhood vaccine uptake as well as comprehend the drivers for vaccine hesitancy, refusal, and delay. Accordingly, the application of Machine Learning and Artificial Intelligence (53) would be beneficial to (a) identify trends, patterns, and prevalence of childhood vaccine uptake and vaccine-preventable illnesses; (b) investigate psychosocial factors and disparities influencing the receipt of vaccines; as well as (c) examine the interface between vaccine-preventable disease outbreaks and vaccine hesitancy/refusal. Specifically, more concerted efforts should be made to implement *Personal Health Libraries* (54) along with *Public Health Observatories* (55) for vaccine acceptance surveillance (56) on a national scale and within the state of Tennessee. These intelligent tools can facilitate precision health promotion to increase vaccination rates (57) as well as examine causal associations between predictors (e.g., SDoH, COVID-19 pandemic policies, etc.) and outcomes (e.g., vaccine uptake, vaccine hesitancy). In addition to facilitating linkages between healthcare systems, these applications could ensure timely access to accurate health information crucial for effective decision-making regarding vaccine access, allocation services, etc. Health policy-driven changes that address vaccine hesitancy, SDoH inequalities, and disparities in vaccination access would be advantageous. Finally, more research that qualitatively examines barriers to vaccine uptake behaviors, as well as drivers to vaccine hesitancy among specific populations, would be beneficial.

## Author Contributions

OO: writing original draft, review and editing, visualization, and conceptualization. RB and RD: review and editing, and conceptualization. AS-N: review and editing, conceptualization, obtained funding, and supervision. All authors contributed to the article and approved the submitted version.

## Funding

This study is partially supported by Grant# 1R37CA234119-01A1 from National Cancer Institute (NCI).

## Conflict of Interest

The authors declare that the research was conducted in the absence of any commercial or financial relationships that could be construed as a potential conflict of interest.

## Footnotes

1 ≥ 4 doses of diphtheria, tetanus toxoid and pertussis, ≥ 3 doses of polio, ≥ 1 measles-containing vaccine, Haemophilus influenza type b full series, ≥ 3 hepatitis b, ≥ 1 varicella and ≥ 4 pneumococcal vaccine.
